# Community-acquired and hospital-acquired respiratory tract infection and bloodstream infection in patients hospitalized with COVID-19 pneumonia

**DOI:** 10.1186/s40560-021-00526-y

**Published:** 2021-01-18

**Authors:** Kirstine K. Søgaard, Veronika Baettig, Michael Osthoff, Stephan Marsch, Karoline Leuzinger, Michael Schweitzer, Julian Meier, Stefano Bassetti, Roland Bingisser, Christian H. Nickel, Nina Khanna, Sarah Tschudin-Sutter, Maja Weisser, Manuel Battegay, Hans H. Hirsch, Hans Pargger, Martin Siegemund, Adrian Egli

**Affiliations:** 1 Clinical Bacteriology and Mycology, University Hospital Basel, Petersgraben 4, 4031 Basel, Switzerland; 2 Department of Biomedicine, Applied Microbiology Research, University of Basel, Basel, Switzerland; 3 Division of Infectious Diseases & Hospital Epidemiology, University Hospital Basel and University of Basel, Basel, Switzerland; 4 Division of Internal Medicine, University Hospital Basel, Basel, Switzerland; 5 Department of Clinical Research, University Hospital Basel, Basel, Switzerland; 6 Department of Intensive Care Medicine, University Hospital Basel, Basel, Switzerland; 7 Clinical Virology, Laboratory Medicine, University Hospital Basel, Basel, Switzerland; 8 Hospital Pharmacy, University Hospital Basel, Basel, Switzerland; 9 Department of Emergency Medicine, University Hospital Basel, Basel, Switzerland; 10 Transplantation & Clinical Virology, Department of Biomedicine, University of Basel, Basel, Switzerland

**Keywords:** COVID-19, SARS-CoV-2, Bacterial secondary infections, Pneumonia, Sepsis

## Abstract

**Objectives:**

SARS-CoV-2 may cause acute lung injury, and secondary infections are thus relevant complications in patients with COVID-19 pneumonia. However, detailed information on community- and hospital-acquired infections among patients with COVID-19 pneumonia is scarce.

**Methods:**

We identified 220 SARS-CoV-2-positive patients hospitalized at the University Hospital Basel, Switzerland (between 25 February and 31 May 2020). We excluded patients who declined the general consent (*n* = 12), patients without clinical evidence of pneumonia (*n* = 29), and patients hospitalized for < 24 h (*n* = 17). We evaluated the frequency of community- and hospital-acquired infections using respiratory and blood culture materials with antigen, culture-based, and molecular diagnostics. For ICU patients, all clinical and microbial findings were re-evaluated interdisciplinary (intensive care, infectious disease, and clinical microbiology), and agreement reached to classify patients with infections.

**Results:**

In the final cohort of 162 hospitalized patients (median age 64.4 years (IQR, 50.4–74.2); 61.1% male), 41 (25.3%) patients were admitted to the intensive care unit, 34/41 (82.9%) required mechanical ventilation, and 17 (10.5%) of all hospitalized patients died. In total, 31 infections were diagnosed including five viral co-infections, 24 bacterial infections, and three fungal infections (ventilator-associated pneumonia, *n* = 5; tracheobronchitis, *n* = 13; pneumonia, *n* = 1; and bloodstream infection, *n* = 6). Median time to respiratory tract infection was 12.5 days (IQR, 8–18) and time to bloodstream infection 14 days (IQR, 6–30). Hospital-acquired bacterial and fungal infections were more frequent among ICU patients than other patients (36.6% vs. 1.7%). Antibiotic or antifungal treatment was administered in 71 (43.8%) patients.

**Conclusions:**

Community-acquired viral and bacterial infections were rare among COVID-19 pneumonia patients. By contrast, hospital-acquired bacterial or fungal infections were frequently complicating the course among ICU patients.

**Supplementary Information:**

The online version contains supplementary material available at 10.1186/s40560-021-00526-y.

## Background

Knowledge about community- and hospital-acquired infections in patients hospitalized with severe acute respiratory syndrome coronavirus 2 (SARS-CoV-2) infection is still limited. In the rapidly growing literature describing the clinical course of coronavirus disease (COVID-19), it has become evident that some patients suffer from acute respiratory distress syndrome (ARDS), systemic inflammation, and a prolonged disease course requiring sustained mechanical ventilation. Accordingly, COVID-19 patients per se would have a high risk of secondary infections [1]. Two recent meta-analyses [2, 3] found that few studies systematically reported frequency of secondary infections in COVID-19 patients and that the microbiological pathogens isolated and diagnostic methods used were most often not described. Overall, 7% of hospitalized COVID-19 patients had a bacterial infection [2], but more than two thirds of patients were treated with antibiotics of which use of broad-spectrum antibiotics was substantial [3]. This paradox emphasizes the relevance of antibiotic stewardship—also in times of a pandemic—to avoid unnecessary empiric antibiotic treatment. Subsequent studies have reported that early bacterial infections are diagnosed in 2–3% of patients overall [4, 5]. By contrast, late bacterial infections are much more frequent and may occur in a third of patients admitted to the intensive care unit (ICU), mainly as ventilator-associated and late onset infections [6]. *Streptococcus pneumoniae* and *Staphylococcus aureus* were the most frequent causes of community-acquired bacterial pneumonia, whereas Enterobacteriaceae and non-fermenters were main causes of hospital-acquired infections [4–6]. Bloodstream infections were reported to occur in around 5% of patients of hospitalized COVID-19 pneumonia patients, which was more frequent than in a comparison cohort [7]. Among 78 patients requiring ICU stay, the 15-day cumulative risk of bloodstream infection was 25% [8]. Moreover, multidrug-resistant bacteria consisted 12% of all isolated pathogens among Spanish COVID-19 patients, stressing the need for microbiological testing in order to direct antibiotic treatment [5].

In this study, we provide detailed information on diagnostics and occurrence of community-acquired and hospital-acquired viral, bacterial, and fungal infections among COVID-19 patients hospitalized at our hospital during the first pandemic wave.

## Methods

In this descriptive study, we identified all consecutive adults with a first SARS-CoV-2-positive test hospitalized at Basel University Hospital during the pandemic phase (from 25 February to 31 May 2020). We excluded patients with elective admissions, who tested positive by routine screening and were asymptomatic, and patients without pneumonia (based on medical chart review documenting absence of respiratory symptoms and/or no pulmonary infiltrates). We further restricted the study cohort to patients with a minimal hospitalization duration of 24 hours. See Supplementary Material for criteria for SARS-CoV-2 testing and polymerase chain reaction (PCR) assay [9].

We then linked microbiology test results performed during the hospitalization, focusing on respiratory tract samples and blood-stream infections.

The University Hospital Basel is tertiary academic care centre in Switzerland with approximately 700 beds and 35,000 annual hospital admissions. The local ethics committee approved the study (EKNZ project ID 2020-00769).

### Additional microbiological diagnostics

We assessed the occurrence of respiratory tract infection and bloodstream infection, by linking microbiological results from respiratory tract samples (including nasopharynx, sputum, tracheal secrete, and bronchoalveolar lavage fluid) and blood cultures taken in COVID-19 patients.

Additional viral diagnostics was performed with BIOFIRE® FILMARRAY® Respiratory *2 plus* Panel PCR to achieve a rapid detection of other relevant viral respiratory pathogens (see Table S1 for examined pathogens) [9].

For the diagnostics of bacterial and fungal pathogens, culture plates including Columbia sheep blood agar (Becton Dickinson GmbH, BD), Colistin Nalidixic Acid blood agar for Gram positive bacteria (BD), and Haemophilus chocolate agar (bioMérieux) and MacConkey (BD) for Gram negatives were inoculated. In the case of additional requests, such as *Legionella* spp. or moulds, specific selective plates for the pathogens were inoculated (BMPA (BD) and Sabouraud Dextrose Agar (BD), respectively). The incubation times and conditions varied for specific pathogens. We used MALDI-TOF mass spectrometry (Bruker, microflex^TM^ or MALDI Biotyper^TM^; MBT 8468 MSP Library, BDAL V9.0.0.0_7854-8468) for identification of bacterial or fungal species. In patients with suspicion of fungal infection, in-house real-time PCR for *Aspergillus* spp. or galactomannan antigen was supplementing culture.

Urine samples were analysed by Sofia® Fluorescent Immunoassay (FIA) for *Streptococcus pneumoniae* antigen and by Quidel Corporation®, San Diego, CA, USA, for the *Legionella pneumophila* serogroup 1.

Blood cultures (aerobic and anaerobic) were incubated for a maximum of 6 days (BacT/Alert® with Virtuo incubators, bioMérieux). The initial pathogen identification was done using MALDI-TOF MS directly from positive culture bottles [10] or BIOFIRE FILMARRAY BCID Panel PCR (bioMérieux, Lyon, France), and after subculturing confirmed using MALDI-TOF MS. For resistance testing, we used VITEK2 (bioMérieux) microdilution, Etest strips (Liofilchem®, Roseto degli Abruzzi, Italiy), or Sensititre® YeastOne® (Thermo Fischer Scientific, Altrincham, UK).

### Community-acquired infection

Viral co-infection was defined as detection of another viral respiratory pathogen concurrent to SARS-CoV-2 diagnosis. Community-acquired bacterial pneumonia was defined as a microbiology-confirmed pneumonia diagnosed concurrent with SARS-CoV-2 infection or within less than 48 h of hospital admission [11]. Community-acquired bloodstream infection was defined as identification of a pathogen from a blood culture taken within 48 h of hospitalization.

### Hospital-acquired infection

Hospital-acquired infection was defined as secondary infection occurring more than 48 h after hospitalization for SARS-CoV-2. Hospital-acquired pneumonia (HAP) and ventilator-associated pneumonia (VAP) were defined as pneumonias occurring 48 h or more after hospitalization or endotracheal intubation, respectively [11]. For possible VAP diagnosis, indicators of worsening oxygenation (FiO_2_ value increase by ≥ 0.20 or PEEP value increase by ≥ 3 cm H_2_O) over 48 h and purulent respiratory secretions and/or a positive culture for a respiratory pathogen was required [12]. ICU patients with clinical signs of a secondary infection (e.g. new fever, increased purulent tracheal secretion, bronchoscopy with purulent or haemorrhagic secretion/aspiration or radiology with consolidations consistent with bacterial pneumonia), but without microbiological evidence, were considered as culture-negative respiratory infections.

We defined hospital-acquired bloodstream infection as identification of a pathogen from a blood culture taken 48 h or more after hospitalization. An infectious disease specialist evaluated all patients with a positive blood culture (at the time of the positive culture) determining the clinical relevance.

### Antibiotic treatment

We retrieved information on antibiotic and antifungal medication administrated during hospitalization from the electronic medical record. Only systemic administrations with duration of more than 24 h were considered.

### Evaluation of ICU patients

Two senior medical doctors (intensive care specialist, MS, and infectious disease specialist, VB) independently performed a re-evaluation of all clinical and microbial findings for ICU patients, in order to identify cases with VAP, tracheobronchitis, and blood-stream infection.

### Statistical analysis

Patients were followed for the occurrence of respiratory tract infections until discharge, death, or end of follow-up (30 June 2020, i.e. a minimum of 30 days), whichever came first. We tabulated gender and median age (with interquartile range, IQR) and calculated frequency of mechanical ventilation, and extracorporeal membrane oxygenation (ECMO). We calculated duration of symptoms before hospitalization and length of stay (median, IQR). We examined the occurrence of co-and secondary infections (pneumonia, tracheobronchitis, and blood-stream infection) and described use of antibiotics and antifungal therapy. Statistical analyses were performed using STATA 12.0 (StataCorp LP, College Station, TX, USA).

## Results

**Fig. 1:**
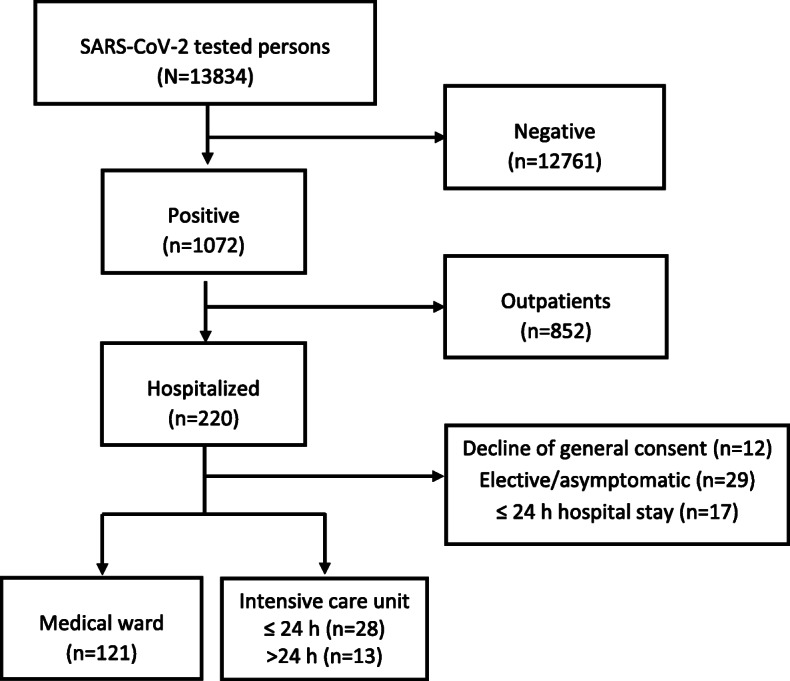
Flowchart depicting the cohort

From 25 February to 31 May 2020, 1073 (7.8%) out of 13,834 persons tested for SARS-CoV-2 at our hospital were positive. Of these, 220 (21%) were hospitalized. Twelve patients declined general consent and were not further considered. We also excluded 17 patients admitted for elective surgery or other medical condition (test positive but asymptomatic), 12 SARS-CoV-2-positive patients without signs of a pneumonia, and 17 patients with a hospitalization duration < 24 h (Fig. 1). Among the remaining 162 patients, median age was 64.4 years (IQR, 50.4–74.2), 99 (61.1%) were males, and median length of stay was 7.7 days (IQR, 4.1–12.3). Median duration of symptoms before hospitalization was 7.6 days (IQR, 4.6–11.5). Among the 162 patients, 41 (25.3%) required ICU admission, and 17 (10.5%) died (Table 1). Characteristics and outcomes of the excluded patients are described in the Supplementary Material.

**Table 1:** Characteristics and outcomes of 162 SARS-CoV-2 hospitalized patients, according to need of intensive care

	All (***n*** = 162)	Non-ICU (***n*** = 121)	ICU-patients (***n*** = 41)
**Age, median (IQR)**	64.4 (50.4–74.2)	62.6 (49.8–74.8)	64.8 (54.7–72.1)
**Males,** ***n*** **(%)**	99 (61.1)	68 (56.2)	31 (75.6)
**Symptom duration before hospitalization, median days (IQR)**^**a**^	7.6 (4.6–11.5)	7.5 (4.1–10.9)	9.2 (6.6–13.3)
**Length of stay, median days (IQR)**	7.7 (4.1–12.3)	6.0 (4.0–9.9)	14.5 (9.5–28.1)
**Mechanical ventilation,** ***n*** **(%)**	34 (21.0)	-	34 (82.9)
**Extracorporeal membrane oxygenation (ECMO),** ***n*** **(%)**	2 (1.2)	-	2 (4.9)
**Antibiotic or antifungal treatment**	71 (43.8)	35 (28.9)	36 (87.8)
**Mortality,** ***n*** **(%)**	17 (10.5)	9 (7.4)	8 (19.5)
**Community-acquired infections,** ***n*** **patients (%)**	6 (3.7)	4 (3.3)	2 (4.9)
Viral co-infection, *n* events (%)	5 (3.1)	4 (3.3)	1 (2.4)
Bacterial pneumonia and bacteraemia, *n* events (%)	1 (0.6)	0	1 (2.4)
**Hospital-acquired infections,** ***n*** **patients (%)**	17 (10.5)	2 (1.7)	15 (36.6)
Bacterial and/or fungal infection, n events (%)	25 (15.4)	2 (1.7)	23 (56.1)
Bacterial infection, *n* events (%)	23 (13.6)	2 (1.7)	21 (51.2)
-Ventilator-associated pneumonia (VAP) ^b, c^	5 (3.1)	0	5 (12.2)
-Tracheobronchitis ^c^	13 (8.0)	0	13 (31.7)
-Non-ventilator-associated pneumonia	1 (0.6)	1 (0.8)	0
-Hospital-acquired bacteraemia	4 (2.5)	1 (0.8)	3 (7.3)
Fungal infection, *n* events (%)	3 (1.9)	0	3 (7.3)
-Ventilator-associated pneumonia (VAP) ^b, c^	2 (1.2)	-	2 (4.9)
-Hospital-acquired candidemia	1 (0.6)	-	1 (2.4)

^a^Symptom duration was unknown for 18 patients

^b^One patient had a multi-microbial VAP with both bacterial and fungal organisms detected in respiratory samples from the event

^c^In two patients with clinically suspected infection, the respiratory samples were negative

### Completeness of microbiology testing

Among the 162 included patients, 87 (53.7%) were examined with BIOFIRE® FILMARRAY® Respiratory *2 plus* Panel, and 35 (21.6%) had one or more respiratory samples for culture. Urinary antigen test for *Legionella pneumophila* and *Streptococcus pneumoniae* was performed in 60 (37.0%) and 48 (29.6%) patients, respectively. Finally, 127 (78.4 %) had one or more blood cultures taken. Completeness of testing was higher for patients with an ICU admission (63.4% respiratory samples for culturing, 68.3% urine antigen for *Legionella pneumophila*, 56.1% urine antigen for *Streptococcus pneumoniae*, and 95.1% had blood culture drawn) (Table 2).

**Table 2:** Completeness of microbiology testing and frequency of positive test result among 162 SARS-CoV-2 hospitalized patients

	All (***n*** = 162)		Non-ICU (***n*** = 121)		ICU-patients (***n*** = 41)	
	Individuals tested, ***n*** (%)	Positive test/tested, ***n*** (%)	Individuals tested, ***n*** (%)	Positive test/tested, ***n*** (%)	Individuals tested, ***n*** (%)	Positive test/ tested, ***n*** (%)
**BIOFIRE® FILMARRAY® Respiratory** *2 plus* **Panel**	87/162 (53.7)	5/87 (5.7)	62/121 (51.2)	4/62 (6.5)	25/41 (61.0)	1/25 (4.0)
**Respiratory culture**^**b**^ **(n = 184 samples)**	35/162 (21.6)	19/35 (54.3)	9/121 (7.4)	4/9 (44.4)	26/41 (63.4)	15/26 (57.7)
***Legionella pneumophila*** **(urinary antigen)**	60/162 (37.0)	0	32/121 (26.4)	0	28/41 (68.3)	0
***Streptococcus pneumonia*** **(urinary antigen)**	48/162 (29.6)	1/48 (2.1)	25/121 (20.7)	0	23/41 (56.1)	1/23 (4.3)
**Blood culture (n = 1110 bottles)**	127/162 (78.4)	20/127 (15.7)	88/121 (72.7)	11/88 (12.5)	39/41 (95.1)	10/39 (25.6)

^a^A positive test result was not equal to clinical infection

^b^Respiratory samples included: tracheal secrete (*n* = 136), sputum (*n* = 25), bronchoalveolar lavage (*n* = 11), and bronchial secrete (*n* = 12)

### Community-acquired infections

Co-detection of other respiratory viral or bacterial pathogens was rare among the 162 hospitalized patients. In five (3.1%) patients, another community-acquired respiratory virus was detected (Table 1 and Table S1). One patient was transferred from another hospital with *Streptococcus pneumoniae* pneumonia and bacteraemia*.*

### Hospital-acquired infections

We observed no hospital-acquired viral infections. However, a total of 14 (8.3%) patients had growth of relevant pathogens in their respiratory samples and by clinical evaluation a secondary bacterial infection (noted in the patient record). The spectrum of pathogens was dominated by gram-negative rods, but importantly also included two *Aspergillus fumigatus* (Table 3). Only one multi-drug resistant pathogen (*Acinetobacter baumannii*, Oxa-23) was isolated in a case transferred from a hospital abroad. Other susceptibility tests were in agreement with the low occurrence of multi-drug resistant pathogens in Switzerland. Median time to infection was 12.5 days (IQR 8–18). Five patients (3.0%) were diagnosed with hospital-acquired bloodstream infection. One patient had *Escherichia coli* bacteraemia (and preceding *Escherichia coli* VAP); one patient had *Pseudomonas aeruginosa* bacteraemia and urinary tract infection; and two patients had catheter-associated sepsis with *Candida albicans* and *Staphylococcus epidermidis,* respectively. Finally, one patient had a *Citrobacter koseri* bacteraemia of unknown origin. Median time to bloodstream infection was 14 days (IQR, 6–30). Additional 15 patients had positive blood cultures that were deemed a contamination (see Table 2 and Table S2 for all microorganism cultured).

**Table 3:** Detected pathogens among patients with clinical signs of respiratory tract infection or bloodstream infection

	All (***n*** = 162)	Non-ICU (***n*** = 121)	ICU-patients (***n*** = 41)
	***n*** (%)	***n*** (%)	***n*** (%)
**Community-acquired pneumonia and bacteraemia,** ***n*** **(%)**	1 (0.6)	0	1 (2.4)
*Streptococcus pneumoniae*^*a*^ (day 1)	1 (0.6)	0	1 (2.4)
**Hospital-acquired pneumonia, n (%)**	1 (0.6)	1 (0.8)	0
*Haemophilus influenzae* (day 6)	1 (0.6)	1 (0.8)	0
**Ventilator-associated pneumonia (among 34 patients), n (%)**	-	-	5 (14.7)
*Acinetobacter baumannii MDR/ Aspergillus fumigatus* (day 20)	-	-	1 (2.9)
*Escherichia coli*^*b*^ (day 7)	-	-	1 (2.9)
*Enterobacter cloacae group*^c^ (day 10)	-	-	1 (2.9)
*Aspergillus fumigatus* (day 13)	-	-	1 (2.9)
Culture negative (day 10)	-	-	1 (2.9)
**Hospital-acquired tracheobronchitis, n (%)**	13 (8.0)	0	13 (39.0)
*Staphylococcus aureus*^d^ (day 3)	1 (0.6)	0	1 (2.4)
*Staphylococcus aureus*^d^*/Klebsiella aerogenes*^*e*^ (day 15)	1 (0.6)	0	1 (2.4)
*Escherichia coli* (day 17)	1 (0.6)	0	1 (2.4)
*Klebsiella aerogenes* (day 12)	1 (0.6))	0	1 (2.4)
*Klebsiella pneumoniae/Proteus mirabilis* (day 18)	1 (0.6)	0	1 (2.4)
*Klebsiella variicola* (day 20)	1 (0.6)	0	2 (4.9)
*Serratia marcescens* ^c^ (day 5 and day 25)	1 (0.6)	0	2 (4.9)
*Pseudomonas aeruginosa* (day 4 and day 13)	2 (1.2)	0	2 (4.9)
*Stenotrophomonas maltophilia*^*e*^ (day 24)	1 (0.6)	0	1 (2.4)
Culture negative (day 4)	1 (0.6)	0	1 (2.4)
**Hospital-acquired bloodstream infection, n (%)**	5 (3.1)	0	4 (9.8)
*Staphylococcus epidermidis* (day 44)	1 (0.6)	0	1 (2.4)
*Escherichia coli*^*b*^ (day 16)	1 (0.6)	0	1 (2.4)
*Citrobacter koseri* (day 4)	1 (0.6)	1 (0.8)	0
*Pseudomonas aeruginosa* (day 8)	1 (0.6)	0	1 (2.4)
*Candida albicans* (day 14)	1 (0.6)	0	1 (2.4)

^a^This patient was diagnosed with blood-stream infection at another hospital and transferred to Basel University hospital

^b^This patient first presented with VAP and later with bacteraemia

^c^This patient had two separate episodes with same pathogen

^d^Methicillin susceptible

^*e*^This patient had two separate episodes with different pathogens

### Antibiotic and antifungal treatment

In total, 71/162 (43.8%) patients received one or more antibiotics or antifungals during the hospitalization. The most frequent antibiotics were amoxicillin with clavulanic acid (*n* = 36, 22.2%), piperacillin/tazobactam (*n* = 30, 18.5%), and ceftriaxone (*n* = 14, 8.6%). In addition, seven (5.8%) patients were treated with an azole or echinocandin (Table 4). In total, 48 patients were treated with one antibiotic only, whereas 23 patients were treated with two or more (up to six) different antibiotics or antimycotics during the hospitalization.

**Table 4:** Antibiotic and antimycotic treatment among 71 of 162 hospitalized SARS-CoV-2 patients

Medication (ATC code)	All (***n*** = 162)^**a**^	Non-ICU (***n*** = 121)	ICU-patients (***n*** = 41)
	***n*** (%)	***n*** (%)	***n*** (%)
**One or more antibiotic or antifungal treatments**	71/162 (43.8)	35/121 (28.9)	36/41 (87.8)
**Penicillins +/- beta-lactamase inhibitors**			
Amoxicillin (J01CA04)	1 (0.6)	0	1 (2.4)
Amoxicillin and clavulanic acid (J01CR02)	36 (22.2)	18 (14.9)	18 (43.9)
Piperacillin/tazobactam (J01CR05)	30 (18.5)	10 (8.3)	20 (48.8)
**Carbapenems**			
Meropenem (J01DH02)	16 (9.9)	1 (0.8)	15 (36.6)
Imipenem (J01DH51)	1 (0.6)	0	1 (2.4)
**Cefalosporins**			
Ceftriaxone (J01DD04)	14 (8.6)	8 (6.6)	6 (14.6)
Cefepime (J01DE01)	4 (2.5)	1 (0.8)	3 (7.3)
Cefazolin (J01DB04)	1 (0.6)	0	1 (2.4)
**Quinolones**			
Ciprofloxacin (J01MA02)	1 (0.6)	1 (0.8)	0
**Sulfonamides with trimethoprim**			
Sulfamethoxazole with trimethoprim (J01EE01)	3 (1.9)	2^b^ (1.7)	1 (2.4)
**Aminoglycosides**			
Tobramycin (J01GB01)	1 (0.6)	0	1 (2.4)
**Polymyxines**			
Colistin (J01XB01)	1 (0.6)	0	1 (2.4)
**Glycopeptides**			
Vancomycin (J01XA01)	2 (1.2)	2^c^ (1.7)	0
**Other antibacterials**			
Daptomycin (J01XX09)	3 (1.9)	0	3 (7.3)
**Antimycotics**			
Fluconazole (J02AC01)	1 (1.2)	0	1 (2.4)
Caspofungin (J02AX04)	3 (1.9)	1 (0.8)	2 (4.9)
Anidulafungin (J02AX06)	1 (0.6)	0	1 (2.4)
Voriconazole (J02AC03)	2 (1.2)	0	2 (4.9)

Only treatment administered more than 24 h was considered as relevant

^a^Several patients received one or more antibiotics

^b^Sulfamethoxazole with trimethoprim was administered as prophylaxis in immunocompromised patients

^c^Vancomycin was administered peroral for treatment of *Clostridioides difficile* infection

### Subcohort of patients admitted to ICU (*n* = 41)

Patients requiring ICU admission for COVID-19 pneumonia had similar median age as the overall cohort, but the proportion of males were higher (75.6% vs. 61.1%). Median duration of symptoms before hospitalization was 9.2 days (IQR, 6.6-13.3), and median length of hospital stay was 14.5 days (IQR, 9.5–28.1). All patients had typical radiological findings of COVID-19 pneumonia at admission. Out of the 41 patients, 34 (82.9%) required mechanical ventilation, and two patients ECMO. Eight patients died (19.5%) during ICU admission. Two patients had co-detection of another respiratory pathogen at time of SARS-CoV-2 diagnosis; one had Parainfluenza virus and another had community-acquired pneumonia and bacteraemia with *Streptococcus pneumonia*. A total of 22 hospital-acquired infections were diagnosed among 16 patients, including five cases of VAP, 13 episodes of tracheobronchitis, and four bloodstream infections (Table 1 and Table 3). Importantly, these infections also covered two invasive *Aspergillus fumigatus* infections. In one patient, *Aspergillus fumigatus* was cultured (tracheal aspirate, day 13), whereas the galactomannan antigen test in blood was negative. However, autopsy confirmed multiorgan invasive aspergillosis. In a second patient, *Aspergillus fumigatus* was cultured (tracheal aspirate, day 20) and confirmed by an Aspergillus-specific PCR (bronchial secrete). Both patients showed a respiratory deterioration before the microbiological diagnosis, and of note, they were not immunosuppressed nor treated with tocilizumab. The majority of patients (*n* = 36, 87.8%) were treated with antibiotic and/or antifungal therapy. Among 24 patients (58.5%) with no clinical signs of bacterial or fungal secondary infections during ICU stay, 19 (79.2%) were treated with antibiotics.

## Discussion

We provide an in-depth analysis of community- and hospital-acquired viral, bacterial, and fungal infections among 162 patients hospitalized with COVID-19 pneumonia at University Hospital Basel, Switzerland. We found that co-infections at time of admission were rare among the patients. By contrast, secondary pulmonary infections and/or bloodstream infections were frequent, in particular among patients requiring ICU admission. Enterobacteriaceae were the most frequent pathogens detected, but also non-fermenters and *Aspergillus fumigatus* were identified causes of pulmonary infection*.*

Several clinical factors have been identified as prognostic factors for poor outcome of COVID-19 [13–15]. However, as demonstrated in two recent meta-analyses [2, 3], few studies systematically reported the frequency of infections in COVID-19 patients, and fewer described the microbiological pathogens or methods used to detect the pathogens. The meta-analyses were based on 28 (*n* = 3448 patients) [3] and 30 studies (*n* = 3834 patients) [2], respectively. Overall, 7% of hospitalized COVID-19 patients had a bacterial infection, with a higher proportion of infections among ICU patients than mixed patients (14% vs. 4%) [2]. Overall, it was unclear whether reported infections were community- or hospital-acquired, and the proportion differed according to the reported method of testing (4% and 10% by culture and unspecified methods, respectively) [3]. Bacterial species were reported in only 13 (*n* = 41 bacteria) and 17 studies (*n* = 27 bacteria), respectively—highlighting the lack of standardized sampling and reporting of bacterial superinfections among COVID-19 patients [16]. Viral co-infections were reported in less than 2% of SARS-CoV-2-positive patients [5, 9]. After publication of the meta-analyses, studies from Spain, France, and the UK have shed some more light onto occurrence of community- and hospital-acquired infections among hospitalized patients [4–6]. In agreement with our findings, viral co-infections and community-acquired bacterial pneumonias were rarely diagnosed (2–3%) [4, 5]. Though based on low absolute numbers, the causative pathogens reported included the usual causes of community-acquired pneumonia such as *Streptococcus pneumoniae*, *Staphylococcus aureus,* and *Haemophilus influenzae*. By contrast, the risk of late bacterial infection seems to exceed a much greater problem among hospitalized patients. Among 989 Spanish COVID-19 pneumonia patients, 73 patients had a microbiologically confirmed infection, counting 74 bacterial infections (e.g. pneumonia, bacteraemia, urinary tract, and intraabdominal infection), seven fungal and seven viral infections. Diagnosis of bacterial infections was performed with urinary antigen tests in 12 patients, and culture in only three patients. The Spanish study identified 15 bacterial and three *Aspergillus fumigatus* hospital-acquired pulmonary infections including 11 VAPs. Among 54 French ICU patients, 20 (37%) had a bacterial pneumonia, of which 15 cases were ventilator-associated and mainly with late-onset [17]. The causative pathogens of hospital-acquired infections included both *Staphylococcus aureus,* as well as different Enterobacteriaceae and non-fermenters, generally known to be frequent courses of VAP [18]. Whereas studies reporting laboratory-detected pathogens found *Mycoplasma pneumonia* to constitute 29% and 42% of pathogens detected (unclear if results were based on serology or PCR), we did not detect any co-infection with these pathogens or *Legionella pneumophila* in our cohort. A US cohort of 5700 patients detected only three patients with *Chlamydia pneumoniae* or *Mycoplasma pneumoniae,* but completeness of testing was unknown [19]. Disseminated aspergillosis cases among COVID-19 patients have been described [20, 21], but the difficulty of the diagnosis also highlighted [22]. In our cohort, one had autopsy-proven disseminated aspergillosis and another suspected aspergillosis (clinical suspicion together with a positive culture and Aspergillus-specific PCR). Both patients showed a respiratory deterioration before microbiological diagnosis of aspergillosis, and none of them were immunosuppressed or received tocilizumab. We found that patients frequently were colonized with *Candida* species, but these were not considered to play a direct role as VAP-causative pathogen [18]. Bloodstream infection may also complicate the clinical course in hospitalized COVID-19 pneumonia patients. Among 227 Danish patients, none had community-acquired bloodstream infection, whereas 12 (5.3%) had a hospital-acquired bloodstream infection. In comparison, the proportion of patients with bloodstream infection was 1.4% in a matched cohort of patients without COVID-19 pneumonia [7]. In an Italian study of 78 critically ill COVID-19 patients, 31 (40%) had at least one bloodstream infection, adding to a cumulative risk of 25% after 15 days of admission [8]. In agreement, we found only one community-acquired bloodstream infection, whereas 10% of ICU patients had a hospital-acquired bloodstream infection. Whereas we found only one multi-drug resistant pathogen, nine multi-drug resistant pathogens were detected in the Spanish cohort [5], stressing the need for microbiological testing in order to guide antibiotic treatment.

Duration of symptoms before hospitalization among patients requiring ICU admission was similar to other hospitalized patients, whereas the excluded patients had shorter symptom duration. Patients requiring ICU admission had a twice as long hospitalization than the overall cohort did, and a high proportion of ICU patients needed mechanical ventilation. Overall, mortality in our study and the Spanish cohort was comparable. The Spanish study concluded that patients with hospital-acquired superinfection had a longer stay and higher mortality (using significance testing) [5]. We refrained from significance testing because question on causality is difficult. Did patients have longer hospitalization durations because of secondary infections, or were secondary infections a consequence of a prolonged hospital duration?

The pooled estimate of supplementary antibiotics among COVID-19 patients was 72% in the meta-analysis (based on 14 studies, *n* = 1689 patients) [3]. The substantial use of antibiotics may reflect that the clinical assessment is complex, especially in the situation of a previously unknown pathogen, where the natural course of, e.g. inflammatory markers is unknown. The fear of bacterial secondary infections may have led to unnecessary empiric broad-spectrum antibiotic therapy. In our cohort, fewer patients than previously reported received antibiotic therapy. Still, not all had culture-proven infections, and importantly, also the majority of ICU patients without clinical signs of bacterial or fungal infections were treated with antibiotics. Overall, empirical use of antibiotics was lower among patients treated at the general medical ward, where procalcitonin was used to guide antibiotic treatment among patients. However, procalcitonin and other inflammatory markers were not relied on in the discrimination between superinfections and cytokine storm among ICU patients, which may explain the substantial overuse of antibiotics in this setting.

Bacterial pneumonia is a frequent complication in patients with respiratory viral infections. For Influenza virus A, up to 35% of patients are diagnosed community-acquired bacterial pneumonia, mainly caused by S*treptococcus pneumoniae* and *Staphylococcus aureus* [23–26]. For COVID-19 pneumonia, the rate of community-acquired bacterial pneumonia seems to be lower. We found a higher occurrence of VAP in our cohort than reported in the Spanish cohort of COVID-19 patients (5 of 41 (12.2%) vs. 11 of 146 (7.5%)), pathogens in both cohorts mainly constituted by gram-negative bacteria. Importantly, *Aspergillus fumigatus* is a serious and non-negligible cause of severe VAP, occurring in around 20% of ICU patients with influenza-associated pulmonary infections [27, 28]. In the Spanish cohort, there were three cases of hospital-acquired infections caused by aspergillosis, and in our cohort two cases. Though it is yet not clear how often aspergillosis complicates the course of COVID-19 pneumonia, several centres have reported cases of disseminated aspergillosis among patients.

A strength of our study is the structured interdisciplinary assessment, combining the microbial findings *with* the clinical presentation. We provide detailed information on the completeness of testing, the laboratory detection of pathogens, and diagnostic criteria. We observed a low rate of community-acquired respiratory viral co-infections, which may be indicative competitive infection situation for viruses targeting the respiratory tract [9]. Atypical bacterial pneumonia was also rare—but is known to most often affect younger adults—and median age in our cohort was 64 years. However, we found that bacterial and fungal secondary infections occurred more often among patients requiring ICU admission than reported in the meta-analysis and the Spanish cohort [5]. The difference may be explained by our restriction to SARS-CoV-2-positive patients with COVID-19 pneumonia (i.e. not only SARS-CoV-2 positive). While most ICU patients had respiratory samples for culture, we may have missed relevant pathogens, when sampling was performed after initiation of antibiotics. In our cohort, the use of antibiotics was lower than reported in the meta-analyses, but still higher than necessary after retrospective evaluation, stressing the need for antibiotic stewardship even in difficult times of a pandemic. Unnecessary antibiotic treatment is crucial to circumvent development of antimicrobial resistance, in addition to other collateral damages such as colonization with resistant pathogens or antibiotic-associated infections, e.g. *Clostridioides difficile*.

## Conclusion

We observed a high frequency of secondary infections among hospitalized SARS-CoV-2-positive patients requiring ICU admission, which complicated the already challenging clinical management of the patients.

## Supplementary Information

**Additional file 1.**

## Data Availability

The data used and analysed for this study are available from the corresponding author on reasonable request.

## Abbreviations

ARDS: Acute respiratory distress syndrome
COVID-19: Coronavirus disease
ECMO: Extracorporeal membrane oxygenation
HAP: Hospital-acquired pneumonia
ICU: Intensive care unit
IQR: Interquartile range
PCR: Polymerase chain reaction
SARS-CoV-2: Severe acute respiratory syndrome coronavirus 2
VAP: Ventilator-associated pneumonia
