# Supplementary material for: Community-acquired and hospital-acquired respiratory tract infection and bloodstream infection in patients hospitalized with COVID-19 pneumonia
Source: J Intensive Care. 2021 Jan 18;9:10. doi: 10.1186/s40560-021-00526-y (PMC7812551; doi:10.1186/s40560-021-00526-y)
Supplement: Supplementary file 1 — Additional file 1. [file 40560_2021_526_MOESM1_ESM.docx]

**Appendix: Supplementary Material**

1. Criteria for SARS-CoV-2 testing and PCR assay
2. Sensitivity analysis of excluded patients
3. Table S1
4. Table S2

*Criteria for SARS-CoV-2 testing and PCR assay*

Patients with acute respiratory disease and/or fever ≥ 38.0 ^○^C, not explained by other disease, were tested for SARS-CoV-2. Screening tests were collected as combined nasopharyngeal and oropharyngeal swab samples. As of end March, all patients admitted to the hospital for any reason were screened at hospitalization. In the first period (from begin until April 2 2020), we used a combination of an in-house developed real-time reverse transcription-PCR detecting the S-gene, and a Roche Test detecting the E-gene[1,2]. Discordant results were confirmed with an in-house test for the N-gene. In the second period (from April 3 and onwards), SARS-CoV-2 assay was performed using Cobas^®^ 6800 (Roche, Rotkreuz, Switzerland) detecting the ORF-1a/b and E-genes[2].

References

1. Goldenberger D, Leuzinger K, Sogaard KK, *et al*. Brief validation of the novel GeneXpert Xpress SARS-CoV-2 PCR assay. J Virol Methods. 2020; 284: 113925.
2. Leuzinger K, Roloff T, Gosert R, *et al*. Epidemiology of SARS-CoV-2 Emergence Amidst Community-Acquired Respiratory Viruses. J Infect Dis. 2020; 222 : 1270-1279.

*Sensitivity analysis of excluded patients*

Among the 46 excluded patients (not covering the 12 patients who declined general consent), median age was 67 (IQR, 36-83), 43% were males, length of hospital stay was 2.1 days (IQR, 0-6), and eight (15.7%) died. Symptom duration until hospitalization was 3.7 days (IQR, 2.7-8.6). Three viral co-infections including human coronavirus NL63, Influenza virus A, and parainfluenza virus 2 were detected. One patient had a positive respiratory culture with *Haemophilus influenza* (detected in bronchoalveolar lavage fluid and bronchial secrete), but had no clinical signs of pneumonia. There were no positive urinary antigen tests among the patients, and no blood-stream infections.

**Table S1 Respiratory pathogens detected by BIOFIRE^®^** **FILMARRAY^®^ Respiratory plus** **Panel (n=87) among 162 SARS-CoV-2 positive hospitalized patients**

|  | **All (n=162)** | **Non-ICU (n=121)** | **ICU-patients (n=41)** |
| --- | --- | --- | --- |
|  | **n (%)** | **n (%)** | **n (%)** |
| **Viral pathogens** |  |  |  |
| Adenovirus | 0 | - | - |
| Coronavirus HKU1 | 1 (0.6) | 1 (0.8) | - |
| Coronavirus 229E | 0 | - | - |
| Coronavirus NL63 | 0 | - | - |
| Coronavirus OC43 | 0 | - | - |
| Coronavirus MERS-COV | 0 | - | - |
| Human Metapneumovirus | 0 | - | - |
| Human Rhinovirus/Enterovirus | 1 (0.6) | 1 (0.8) | - |
| Influenza A virus | 1 (0.6) | 1 (0.8) | - |
| Influenza B virus | 0 | - | - |
| Parainfluenza 1 | 0 | - | - |
| Parainfluenza 2 | 0 | - | - |
| Parainfluenza 3 | 1 (0.6) | - | 1 (2.3) |
| Parainfluenza 4 | 0 | - | - |
| Respiratory Syncytial Virus* | 1 (0.6) | 1 (0.8) | - |
|  |  |  |  |
| **Bacterial pathogens** |  |  |  |
| *Bordetella parapertussis* | 0 | - | - |
| *Bordetella pertussis* | 0 | - | - |
| Mycoplasma pneumoniae | 0 | - | - |
| Chlamydia pneumoniae | 0 | - | - |

**Table S2 All microorganisms cultured among 162 SARS-CoV-2 positive hospitalized patients**

|  | **Culture (n)** | **All (n=162)** | **Non-ICU (n=121)** | **ICU-patients (n=41)** |
| --- | --- | --- | --- | --- |
|  |  | **n (%)** | **n (%)** | **n (%)** |
| **Respiratory samples^b^ (n=35 tested)** |  |  |  |  |
| **Gram positive bacteria** |  |  |  |  |
| *Corynebacterium jeikeium* | 1 | 1 (0.6) | 0 | 1 (2.4) |
| *Enterococcus faecalis* | 2 | 1 (0.6) | 0 | 1 (2.4) |
| *Staphylococcus aureus*^c^ | 5 | 3 (1.9) | 1 (0.8) | 2 (4.9) |
| *Staphylococcus epidermidis* | 4 | 3 (1.9) | 0 | 3 (7.3) |
| *Streptococcus anginosus group* | 1 | 1 (0.6) | 0 | 1 (2.4) |
| *Streptococcus pneumoniae* | 1 | 1 (0.6) | 0 | 1 (2.4) |
| **Gram negative bacteria** |  |  |  |  |
| *Acinetobacter baumanii, MDR* | 1 | 1 (0.6) | 0 | 1 (2.4) |
| *Citrobacter koseri* | 1 | 1 (0.6) | 0 | 1 (2.4) |
| *Enterobacter cloacae group* | 3 | 1 (0.6) | 0 | 1 (2.4) |
| *Escherichia coli* | 5 | 3 (1.9) | 0 | 3 (7.3) |
| *Haemophilus influenza* | 1 | 1 (0.6) | 1 (0.8) | 0 |
| *Klebsiella aerogenes* | 2 | 2 (1.2) | 0 | 2 (4.9) |
| *Klebsiella pneumonia* | 9 | 1 (0.6) | 0 | 1 (2.4) |
| *Klebsiella variicola* | 9 | 2 (1.2) | 0 | 2 (4.9) |
| *Proteus mirabilis* | 7 | 1 (0.6) | 0 | 1 (2.4) |
| *Pseudomonas aeruginosa* | 12 | 3 (1.9) | 1 (0.8) | 2 (4.9) |
| *Serratia marcescens* | 2 | 1 (0.6) | 0 | 1 (2.4) |
| *Stenotrophomonas maltophilia* | 4 | 1 (0.6) | 0 | 1(2.4) |
| Gram negative rods unspecified | 8 | 4 (2.5) | 2 (1.7) | 2 (4.9) |
| **Yeast and moulds** |  |  |  |  |
| *Candida albicans* | 13 | 6 (3.7) | 1 (0.8) | 5 (12.2) |
| *Candida dubliniensis* | 2 | 1 (0.6) | 0 | 1 (2.4) |
| *Candida* species | 8 | 5 (3.1) | 0 | 5 (12.2) |
| *Aspergillus fumigatus* | 3 | 2 (1.2) | 0 | 2 (4.9) |
| Mould unspecified | 5 | 4 (2.5) | 3 (2.5) | 1 (2.4) |
| **Blood culture^a^ (n=127 tested)** |  |  |  |  |
| *Bacillus cereus* | 2 | 1 (0.6) | 1 (0.8) | 0 |
| *Candida albicans* | 5 | 1 (0.6) | 0 | 1 (2.4) |
| *Citrobacter koseri* | 3 | 1 (0.6) | 1 (0.8) | 0 |
| *Cutibacterium acnes* | 1 | 1 (0.6) |  | 1 (2.4) |
| *Escherichia coli* | 1 | 1 (0.6) | 0 | 1 (2.4) |
| *Pseudomonas aeruginosa* | 1 | 1 (0.6) | 0 | 1 (2.4) |
| *Staphylococcus auricularis* | 1 | 1 (0.6) | 1 (0.8) | 0 |
| *Staphylococcus capitis* | 1 | 1 (0.6) | 1 (0.8) | 0 |
| *Staphylococcus epidermidis* | 14 | 9 (5.6) | 4 (3.3) | 5 (12.2) |
| *Staphylococcus hominis* | 8 | 4 (2.5) | 2 (1.7) | 2 (4.9) |
| *Streptococcus pneumoniae^b^* | Unknown | 1 (0.6) | 0 | 1 (2.4) |

^a^15 patients had positive blood cultures that were deemed to be contaminations (*Bacillus cereus*, n=1;

*Cutibacterium acnes*, n=1; *Staphylococcus auricularis*, n=1; *Staphylococcus capitis*, n=1; *Staphylococcus epidermidis,* n=9; *Staphylococcus hominis,* n=4). Two of these patients had poly-bacterial contaminations. ^b^ This patient was diagnosed with blood-stream infection at another hospital and transferred to Basel University hospital. ^c^ Respiratory samples included tracheal secrete (n= 136), sputum (n=25), bronchoalveolar lavage (n=11), and bronchial secrete (n=12). The following bacteria were cultured, but not considered relevant (n=number samples): *Corynebacterium jeikeium* (n=1), *Enterococcus faecalis* (n=2), *Staphylococcus epidermidis* (n=4), candida species (n=23), and mould unspecified (n=5). A total of 21 samples were negative, and 52 had growth of normal mouth flora registered. ^d^ all *Staphylococcus aureus* isolates were methicillin susceptible.
